# Factors that shape the successful implementation of decommissioning programmes: an interview study with clinic managers

**DOI:** 10.1186/s12913-021-06815-4

**Published:** 2021-08-12

**Authors:** Inga-Britt Gustafsson, Ulrika Winblad, Lars Wallin, Mio Fredriksson

**Affiliations:** 1 Department of Public Health and Caring Sciences, Health Services Research, Uppsala University, Uppsala, Sweden; 2 Dalarna County Council, Falun, Sweden; 3 Centre for Clinical Research, Falun, Dalarna Sweden; 4 School of Education, Health and Social Studies, Dalarna University, Falun, Sweden

**Keywords:** Decommissioning, Leadership, Clinic manager, Healthcare professionals, Implementation, Local healthcare organisation

## Abstract

**Background:**

As a response to many years of repetitive budget deficits, Region Dalarna in Sweden started a restructuring process in 2015, and implemented a decommissioning programme to achieve a balanced budget until 2019. Leading politicians and public servants took the overall decisions about the decommissioning programme, but the clinical decision-making and implementation was largely run by the clinic managers and their staff. As the decommissioning programme improved the finances, met relatively little resistance from the clinical departments, and neither patient safety nor quality of care were perceived to be negatively affected, the initial implementation could be considered successful. The aim of this study was to investigate clinic managers’ experience of important factors enabling the successful implementation of a decommissioning programme in a local healthcare organization.

**Methods:**

Drawing on a framework of factors and processes that shape successful implementation of decommissioning decisions, this study highlights the most important factors that enabled the clinic managers to successfully implement the decommissioning programme. During 2018, an interview study was conducted with 26 clinic managers, strategically selected to represent psychiatry, primary care, surgery and medicine. A deductive content analysis was used to analyze the interviews. By applying a framework to the data, the most important factors were illuminated.

**Results:**

The findings highlighted factors and processes crucial to implementing the decommissioning programme: 1) create a story to get a shared image of the rationale for change, 2) secure an executive leadership team represented by clinical champions, 3) involve clinic managers at an early stage to ensure a fair decision-making process, 4) base the decommissioning decisions on evidence, without compromising quality and patient safety, 5) prepare the organisation to handle a process characterised by tensions and strong emotions, 6) communicate demonstrable benefits, 7) pay attention to the need of cultural and behavioral change and 8) transparently evaluate the outcome of the process.

**Conclusions:**

From these findings, we conclude that in order to successfully implement a decommissioning programme, clinic managers and healthcare professions must be given and take responsibility, for both the process and outcome.

**Supplementary Information:**

The online version contains supplementary material available at 10.1186/s12913-021-06815-4.

## Background

Healthcare systems around the world face major challenges in managing growing costs for healthcare services, and publicly financed healthcare systems, in particular, need to prioritise among the range of services that citizens expect to access [1]. Hence, decommissioning in healthcare organisations has received increased attention in the research literature and has been referred to as the removal, reduction and replacement of healthcare services and interventions, which may result in re-evaluating existing services, thereby generating space for services that are more effective and beneficial for patients [2]. It is a broader concept than, for example, de-implementation that focuses on “stopping practices that are not evidence-based” [3, 4]. However, the evidence base is weak regarding how to support healthcare professionals, including clinic managers, when implementing decommissioning decisions [2, 5]. In previous research, healthcare professionals infer that the lack of experience and a `blueprint’ on best practice in how to carry out decommissioning activities lead to confusion over roles and responsibilities [2].

That policies such as decommissioning programmes are complex and made even more complicated when they are put into action has been confirmed in several studies [6, 7]. Although healthcare professionals report a knowledge-gap regarding decommissioning activities, studies have been carried out with results that can be useful in decommissioning processes. For instance, it has been shown that it is important to present and frame the need for change in a credible way in order to succeed with complex changes, like a decommissioning programme in a politically and culturally acceptable manner [8]. To facilitate the process and establish the need for change, a process of local sense-making is required to take place when old assumptions of realities of best practice are reconciled with new realities and evidence [9]. This process creates challenges for those involved, and organisations and healthcare professionals need to create their own story to get a shared image and to tackle challenges for how to carry out the decommissioning processes. To create a shared image, the executive leadership team requires communication skills to unify and link together different levels in the organisation, in order to create participation, acceptance and credibility [7, 10, 11].

During decommissioning processes that involve major organisational changes, the executive leadership team, and in particular clinical champions, are the most important key influencers, who with their experience, capacity and integrity, have the ability to prioritise the organisation’s overall objectives [12]. Furthermore, the executive leadership team and the clinical champions need to predict and respond proactively to the resistance that is to be expected when the decommissioning processes infringe on local clinics and clinic managers [12]. Thus, to succeed with decommissioning in local healthcare organisations it is wise to address the local context, strengthen commitment and clarify expected results [13]. To facilitate the implementation of decommissioning, international experts identified best practice in a framework with three categories: change management and implementation; evidence and information; and relationships and political dimensions. In the present study, this framework [4] was used to analyse the clinic managers’ experiences of successfully implementing a decommissioning programme in a local healthcare organisation in Sweden, thus illustrating success factors in an empirical case.

### THE CASE - decommissioning processes in region Dalarna

Sweden has 21 regions, which are politically governed and responsible for providing healthcare to the citizens of the region. Region Dalarna has more than 8500 employees providing services to 281,000 inhabitants in 15 municipalities. Compared to the other 20 regions in Sweden, the overall health status of the population in Dalarna is relatively poor.

A decommissioning process in Region Dalarna started in 2015. Until 2019, 700 million SEK needed to be cut to achieve a balanced budget. The decommissioning process included many aspects that were formulated into a number of restructuring plans: e.g., the concentration of services to more urban areas, efficiency improvements, changed staffing policies, closing down a rehabilitation centre, training pools and an ambulance station. During the decision-making process that led to the restructuring plans, the executive leadership team and all clinic managers got access to a review report regarding the region’s economic situation, staffing, quality of care and other central indicators, in which comparisons with other regions were made and potential decommissioning areas pointed out.

At the same time, during 2015, Region Dalarna’s healthcare organisation was divided into four divisions: psychiatry, primary care, surgery and medicine. These division managers heading the new divisions had up to that time worked as clinic managers in Region Dalarna and were perceived as highly qualified physicians and were well respected among staff (clinical champions). Throughout the decommissioning process, the division managers represented medical expertise, with the capacity to execute and implement horizontal priorities within the divisions. The executive leadership team (referred to as the region director, deputy region director, chief financial officer and chief medical adviser) was also reinforced by the division managers as well as by analytical and legal expertise. Additionally, the division and clinic managers had support to obtain a knowledge base and evidence in medical priority setting issues, and occasionally consulted a local expert group that worked with health technology assessment.

### Successful decommissioning processes

Overall, an evaluation showed that nearly 150 (almost 95%) of the decommissioning activities in the restructuring plans decided in 2015 had been implemented by the end of 2016 [14]. Furthermore, this evaluation indicated that neither patient safety nor the quality of care had been negatively affected by the decommissioning programme, and there was a stable positive trend since 2014 in quality of care for the division’s primary care, surgery and medicine [15]. The data reported in 2016 showed an improvement in the region’s economy, and the fact that the restructuring plans met relatively little resistance from clinics and clinic managers indicated that the decommissioning process was successful in 2015–2016 [14, 16]. Also, the employee survey in Region Dalarna indicated improved results in comparison to the survey before the start of the decommissioning programme, and in comparison with other regions in Sweden, it showed better results regarding the employees’ perception of work motivation and the region’s leadership [17]. In summary, most of the decommissioning programmes’ goals seemed to be achieved by the of 2016 [14–16].

### Clinic managers and their role in decommissioning processes

Healthcare professionals, and particularly physicians, often disagree with politicians and public administrators on how to carry out improvement and development efforts. This implies that what they perceive to be organisationally and not professionally initiated changes have little chance of being successful [18]. A recent study has shown that clinic managers act as the primary implementers of decommissioning programs in local health care organisations. The clinic managers experience of economic governance and their important role as the ones with the ability to bridge and handle professional culture, motivate and soften the negative consequences of staff-level changes is crucial. In addition, clinic managers must communicate the financial constraints to enable the healthcare professionals to contribute during the decommissioning activities, for example, by recommending potential clinical-led changes, evaluating feasibility and impact on the quality of care, as well as professional responsibility [19]. While implementing the decommissioning programme in Region Dalarna, the overall goal was to achieve a balanced budget without compromising patient safety and quality of care. According to the law, clinic managers must ensure the patient’s safety, as well as continuity and coordination of care [20]. The clinic managers must also adhere to national ethical guidelines for priorities, i.e., the principles of human dignity, need and solidarity, and cost-effectiveness. These guidelines aim to ensure that a greater proportion of resources are allocated to the care of those in greatest need [21].

Clinic managers act in the complex context described above, and in order to contribute with a more comprehensive understanding of the challenges clinic managers (the primary implementers) face during decommissioning processes, it is crucial to empirically investigate their experiences. This study attempts to develop a better understanding of how decommissioning programmes unfold, which, as suggested, is a crucial step towards improving decommissioning policy and practice [2].

### Aim of the study

The aim was to investigate clinic managers’ experiences of successfully implementing a decommissioning programme in a local healthcare organization and what factors they considered as the most important enabling the decommissioning process.

## Methods

### Study design

A qualitative interview study.

### Setting and sample

The strategic selection of respondents was based on all 50 clinic managers from the four divisions: psychiatry, primary care, surgery and medicine (see Table 1). The clinic managers were selected based on being responsible for patient care and decisions regarding treatment and medical interventions, and thus, costs linked to patient care. This selection meant that service departments such as x-ray clinics and emergency departments were excluded. Another inclusion criterion was clinic managers must have worked in the region since 2016. In primary care, respondents were selected based on an ambition to cover different geographical areas in Region Dalarna and of belonging to primary care units that carried out major changes during 2015–2017. Twenty-seven clinic managers were invited to participate in the study via email and informed about the study’s purpose, and that participation was voluntary. Twenty-six clinic managers decided to participate. Prior to the interviews, the participants were again informed both verbally and in writing about the study’s purpose, including the voluntary nature of the study and the possibility to withdraw at any time. When deciding to participate, the clinic managers signed their consent. The study was approved by the regional ethics board in Uppsala (No. 2016/504).

**Table 1:** Characteristics of the clinic managers (*n* = 26)

Division	Medicine (***n*** = 8)	Primary care (***n*** = 4)	Psychiatry (***n*** = 6)	Surgery (***n*** = 8)
Gender, *n*				
Female	7	4	3	2
Male	1		3	6
Outpatient care clinics		4	3	
Specialised care clinics	8		3	8

### Data collection

The interviews were carried out by the first author. A semi-structured interview guide was developed by the research team, based on retrenchment (cutting expenses in public services), management and implementation literature. The questions were designed to capture how the respondents experienced their participation in the decommissioning process. In particular, the questions focused on the clinic managers’ experiences of the decision-making process leading to the restructuring plans, their efforts to involve the profession and how they obtained acceptance for the plans at the clinical departments, as well as their role in the implementation. Questions in the guide were for example: ‘How would you describe your work situation during these last three years?’ and ‘Do you have any examples of decisions that you have made that have been crucial to the success of your work?’, ‘Do you have any examples of decisions you regret that made the job more difficult?’ and ‘What have been the most important factors that enabled the decommissioning process?’ The interviews lasted 30–75 min, were audiotaped with permission and conducted at the clinic managers’ workplaces from January to June 2018.

### Analysis procedure

The interviews were transcribed verbatim and analysed through deductive content analysis guided by a structured analysis matrix [4, 22, 23], i.e., the framework developed by Robert et al. The deductive approach was chosen because there is prior research about factors that facilitate the successful implementation of decommissioning that would benefit from further description [22, 24]. By organising the collected data into the analysis matrix, our data could be compared with the categories in the framework and our study is thus an empirical exploration or validation of the framework as well, which Robert et al. have suggested as a next step. The content of the interviews was largely gathered around eight factors in the framework, which became clearer with the number of interviews conducted. The number of respondents seemed to be sufficient as the text from the interviews was very rich in content and no new themes emerged from the latter interviews.

First, the interviews were read through several times by the first and last author to get a sense of the wholeness. Second, the first author used Nvivo 10.0 to code relevant material into categories and subcategories according to the framework. The coded material stayed as quotes in the subcategories throughout the process. Third, the last author read the subcategories and verified the findings to secure reproducibility [25]. A few differences in the subcategories coding were resolved through ongoing discussion to achieve consensus and precision. Fourth, all authors read and discussed three interviews to ensure dependability and confirmability. Fifth, from the subcategories that contained the most material, the clearest descriptive quotes were selected to be included in the results.

### The framework

The framework is based on factors believed to shape the successful implementation of decommissioning decisions [4]. Through three Delphi rounds, 30 international experts participated in the development of the framework and contributed with opinions on what should, and what really shapes decommissioning processes. The experts agreed on three categories that ought to inform decommissioning processes: quality and patient safety, clinical effectiveness and cost-effectiveness. They also identified a discrepancy between these ideals and the factors that really influenced the decisions in practice, and thus grouped best practice in decommissioning into three themes: change management and implementation, evidence and information, and relationships and political dimensions. These three categories and underlying factors (subcategories), described as shaping the extent to which decommissioning was implemented as planned, is presented in descending order of importance within each category (Table 2).

**Table 2:** Framework by Robert et al. (2014) rating factors in descending order, in terms of importance within each category, in shaping the extent to which decommissioning is implemented as planned

Factor	
***Change management and implementation strategy***	
• Strength of executive leadership	
• Strength of clinical leadership	
• Quality of communication	
• Clarity of specific aims and objectives at start	
• Extent of cultural and behavioral change	
• Attention throughout to human aspects of process of change	
• Quality of project management	
• Availability of resources to support decision-making and implementation processes	
• Quality of strategic planning	
• Training and preparation of staff	
• Clarity of incentives and levers to support change	
• Complexity of decommissioning programme	
• Pace of change	
***Evidence and information***	
• Demonstrable benefits	
• Clarity of evidence/data to support business case, ongoing monitoring and impact assessment	
• Clarity around new patient pathways	
• Review/evaluation of process	
• Availability of alternative services	
• Extent of adoption elsewhere of new intervention/service	
***Relationships and political dimensions***	
• Clarity of rationale/case for change	
• Nature and extent of clinician engagement/involvement	
• Level of political support	
• Transparency of decision-making process	
• Nature and extent of patient/public engagement/involvement	
• Quality of partnership working with relevant agencies	
• Extent to which challenges vested interests	
• Nature and extent of media coverage	
• Stability within the local health economy during transition	
• Reputation of existing providers	
• Meets community expectations	

## Results

In the interviews, the respondents shared experiences of their participation in the decommissioning programme in the local healthcare organisation. The clinic managers’ experiences illustrated factors that enabled the implementation at their clinics. About three quarters of the 30 factors (subcategories) in the framework were identified in the interviews. The categories of change management and implementation contained the most material (quotes). Eight of the factors, which we focus on in the results section, were more pronounced by the clinic managers and consistently highlighted as crucial in order to succeed with the decommissioning programme: 1) clarity of rationale for change, 2) strength of executive leadership, 3) strength of clinical leadership, 4) clarity of evidence/data to support the business case, 5) attention throughout to human aspects of the process of change, 6) demonstrable benefit, 7) the extent of cultural and behavioural change, and 8) review/evaluation of the process.

### Change management and implementation strategy

#### Strength of executive leadership

All clinic managers expressed the ‘strength of executive leadership’ as being very important. The clinic managers described the leadership of Region Dalarna under this period as stronger, clearer and more resolute in contrast to previous decommissioning attempts in the region. The division managers (the clinical champions) were considered invaluable in creating the necessary hope and confidence in how to execute the decommissioning programme in a professional way, and with worthiness towards everyone involved. One of the clinic managers said:

’….it is all about having faith and trust in the managers above you, ... I think that the division managers we have today, as they all have a long, long experience within our profession and are very knowledgeable, have been the key to the success we see today…persons that one has a lot of confidence in due to their knowledge.‘

The division managers’ expertise in issues such as insights into the limited resources of the region, upcoming new expensive treatment options and increased costs of healthcare, enabled them to argue for the need of a new approach among the clinic managers. An experienced clinic manager described it like this:

‘This was the best cost-cutting programme I have ever so far experienced, as to my knowledge. The top leadership team with the division managers, all took the lead and they let us clinic managers be a part of all this. No general cost-cutting by lowering cost levels at all units by the same percentage…. We were all gathered, and yes, I think this was proof of very strong leadership demonstrated by the division managers.‘

However, there were also other opinions, and some of the clinic managers pointed to the importance of fairness between clinics and saw a problem in that every clinic manager did not equally contribute to the hard work of balancing the challenging financial situation. Some of the clinic managers felt that there should be sanctions for those clinic managers who did not contribute, expressed by a clinic manager as:

’…I have to look at this from the perspective of fairness… there are few things that make people so very angry and unwilling to participate.…this is regardless of whether it is about the clinic or about individuals.‘

According to the clinic managers, and similar to their own experience, fairness was crucial to achieve compliance to all decisions at the clinics. One clinic manager recalled this situation:

‘The region director asked: How do you think we should handle clinic managers who do not comply with decisions taken? There was a wise person, who immediately then responded: Treat that person as anyone else. First a warning and then you lose your job. And this has not been the norm before. This is [unfairness], in my view, how people completely lose faith and do not want to stay at work.‘

#### Strength of clinical leadership

In the fine art of balancing the execution of cost-cutting plans and involving the staff, the strength of the clinical leadership was commented by one of the clinic managers as*:* ‘… this is what it is all about. You have to get all your staff on board. This is really the art of leadership‘. One clinic manager described the satisfaction of finally having full control of the financial budget:

‘Just the fact that I now own a budget covering all activities we are responsible for… makes me feel that I am empowered to make informed decisions. Previously, realise that, I could hardly make any decisions at all by myself, as I knew that my budget had an annual deficit of 15 million. That is quite a “show-stopper”.’

The formation of the four divisions, i.e., psychiatry, primary care, surgery and medicine, created a team feeling between the clinic managers within each division. The team feeling eased and strengthened the discussions on the fair distribution of resources between clinics and departments that the division and clinic managers had to carry out. The possibility of support between colleagues in the divisions contributed to creating a ‘strength of clinical leadership’ and was considered to be of great value. However, a number of clinic managers reported that the changes they made at their clinics sometimes challenged other clinic managers. To infringe on other professionals’ autonomy was perceived by a few clinic managers as a very provocative measure.

‘I also had to face a situation when colleagues got very irritated, and other clinic managers were irritated with me for wanting to use the doctors in a different way. …they meant that the doctors need to decide by themselves when to work, what to work on and so forth, but never tell them too much what to do. Yes, there was some initial resistance when I started…., but now, nobody wants to go back to the old system.’

#### The extent of cultural and behavioural change

Several clinic managers worked hard to increase the awareness of cost efficiencies and to achieve a better understanding of the consequences of making decisions that could increase costs at their clinics. Regular discussions at the clinic ensured that the decision-making about expensive treatments was based on evidence and defensible in relation to the needs of other patient groups. The clinic managers described their desire to create a different approach and a ‘cultural and behavioural change’*,* in particular among the physicians at the clinics. In this way, resources could be spent in a more cost-efficient way and be justified from an ethical perspective. When meeting a patient, it can sometimes be hard to keep in mind that the resources are limited. One of the clinic managers gave a plausible scenario at a clinic:

‘Right now I have this patient in front of me. I have to make all those decisions that I feel are relevant and necessary to make things as good as possible. In that situation, one does not take into consideration the financial implications, so this is a very hard process to communicate‘.

A clinic manager confirmed a new attitude among clinic managers towards the region’s strained financial situation:

’…today there is more awareness, before it was more loose and vague. There was a culture that if you spent more than what is in your budget…there will be new fresh money there next year. This is definitely not how people think today‘.

#### Attention throughout to human aspects of the process of change

Most clinic managers mentioned a lot of work, close involvement, and tough emotional situations to deal with in order to secure ‘attention throughout to human aspects of the process of change’. Nevertheless, it was perceived as satisfying to take an active part in the process, and most clinic managers described the process as a boost for their own personal development. A clinic manager said:

‘… how exposed one is as a leader having to play this role, how questioned I have been and how I have just been lying on the couch in the kitchen, cried and told my partner that I am not the right person to do this, I cannot cope with it, no energy left. I have been so frustrated. It has been very hard at times, but at the same time ….I feel that I have learnt a lot. The entire management group has grown as we executed our task, but it has been demanding.‘

Incorporating the region’s need to save money with the interests, desires and feelings of the staff involved, required dedicated managers who invested a great deal of themselves in the clinical leadership. The clinic managers described strong feelings among both employees and themselves. The need for comfort during the decommissioning process was felt by employees as well as by the managers, and a clinic manager handled the situation like this:

‘I had to take long walks with staff members, who lost their jobs and somehow, we both got back on the track. Facing these realities was almost unbearable. I am a very sensitive and emotional person, which I actually could also use in this process. I needed to comfort and I wanted to be close to many of the staff members. We all had a tough time.‘

The challenging decisions about removal and concentration of healthcare services to more urban areas affected employees and even forced some of them to change both workplace and place of residence. These decisions were demanding, and a clinic manager described the importance of ‘attention throughout to human aspects of the process of change’ and the value of sharing emotions with others being involved:

‘It has been about being a PART of the staff. To be allowed to cry together with them. I think that has been important. Then everyone knows that also I am suffering from what is happening. I am not untouchable and above everyone else.‘

### Evidence and information

#### Demonstrable benefits

Many clinic managers used the decommissioning programme as an opportunity to strengthen the competence of the work teams and to deal with the staff shortage situation that affected the whole region. The clinic managers described ‘demonstrable benefits’ by reporting about how quality had improved when services had been coordinated to fewer places. One clinic manager pointed out:

‘We have now been able to build a fantastic health service here. We would never have been able to achieve this, if we had stayed geographically spread out.‘

Another colleague gave one more example of demonstrable benefits:

‘We are making efforts to ensure that access to health care is reasonable throughout in Region Dalarna and that things are done in a more uniform way. This we did not have before.‘

#### Clarity of evidence/data to support the business case

The clinic managers felt that the review report, regarding the region’s economic situation, staffing and quality of care, facilitated the process and generated a trustworthy starting point. All respondents perceived the decision-making process leading to the decommissioning programme characterised by integrity and the review report as ‘clarity of evidence’ crucial to inform choices about how to achieve the best health improvement outcomes and to back up the need for change. The review report was also important in the discussions with clinic managers that could potentially obstruct the process. Several clinic managers expressed themselves similarly to this colleague:

’…I really do think that I have gained many new insights and most of it has been very good. The review report prior to our discussions was very good. It was excellent to gather all the clinic managers in order for us to come up with various suggestions.‘

As the report contained comparisons to other regions, Region Dalarna could be evaluated in a national context, and a clinic manager asserted:

‘We were different in comparison with other regions. We realised that it is not reasonable to provide high-quality specialist health care at so many different locations. We simply cannot do that.’

#### Review/evaluation of process

Clinic managers emphasised the importance of newly established methods for follow-ups, ‘review and evaluation of the process’ and analysis in relation to the ongoing decommissioning process. One clinic manager explained it this way:

‘You are being watched today in an entirely new way, and you are “seen” all the time, month by month. This was not the case before*.* So, today everyone is being scrutinised. This has also resulted in a situation when everyone has a “watch” on everyone else, which never was done before.‘

Another clinic manager pointed out the fact that there is now an improved trust in the ability of the region to handle crises:

‘We are such a huge organisation, so when each and everyone makes a small adjustment, then it can result in big things. Just this I thought was very important. Then we noticed down the road, three years have now passed, that it really has changed things and then you believe even more in this approach.’

### Relationship and political dimensions

#### Clarity of rationale/case for change

In general, the clinic managers gave very positive feedback about the ´clarity of rationale for change´ and how the decommissioning process was initiated. This time, the executive leadership team managed to convey the message that the situation was serious. The clinic managers were gathered, all physically together, at several occasions and received extensive material, including the review report that shed light on the region’s difficult situation. The region had struggled with the financial situation previously, but never been as transparent as now. When the region had to borrow money for staff salaries, the clinic managers felt that everyone realised that the financial situation was at a critical point. One clinic manager clarified:

‘It is quite obvious, one may think, that there is something very wrong when you have to borrow money to cover the expenses for salaries. One does not, probably, need more than this as a “message” about the situation.‘

As the executive leadership team managed to clarify the severity of the situation, the clinic managers started to get the picture, and a ‘rationale for change’ with a common responsibility to the future of Region Dalarna was established. A clinic manager expressed that he felt that the *‘*head in the sand behaviour‘ had to stop and another one recalled: ’…now is the time, we must all give a hand, this is about rolling up our sleeves and start to work‘. Furthermore, another clinic manager described new insights and defined the ‘clarity of rationale for change’ and the critical financial situation as:

‘It is good to review our current practice, driven by the fact that we have a tough financial situation. Then, one must really, really scrutinise things that we do. This is the point for different reasons. One cannot go on walking in your “used old shoes”, there is now a true incentive to review and question everything we do. The reason is that the money is no longer there for us‘.

## Discussion

In order to succeed with complex changes such as decommissioning activities, Robert et al. previously identified a number of factors [4]. The current study highlighted that the clinic managers considered eight of these factors crucial in their efforts to implement a decommissioning programme. Based on these findings, recommendations for successful implementation would suggest, at first, that it is necessary to create a shared image of the rationale for change by using evidence to frame the problem in a credible way. Furthermore, the processes of deciding on decommissioning activities must be based on evidence, with the clear goal of not compromising quality, patient safety and ethical responsibility. Another important factor is to involve clinic managers from an early stage to take responsibility for the process and results in order to legitimise the process. Furthermore, it is crucial to secure an executive leadership team represented by clinical champions to ensure that the organisation’s overall goals and objectives are achieved. In addition, all managers of the organisation have to be prepared to handle a process characterised by tensions, resistance and strong emotions. Along the way, there is a need to communicate demonstrable benefits as well as transparently evaluate the outcome to achieve a fair process.

The executive leadership team managed to communicate the seriousness of the economic situation and the rationale for change became clear to the clinic managers. The review report, with comparisons to other Swedish regions, clarified that many other regions were shown to have more cost-effective organisations. Similar arguments pointed out the importance of presenting and framing the rationale for change in a credible way, has been put forward previously by researchers [8, 26]. The clinic managers in the study felt it had been a fair and inclusive process, with extensive participation, transparent, knowledge-driven and professionally controlled. They expressed, regardless of professional background, that their professional skills, knowledge and commitment had been encouraged by the executive leadership team. These statements are similar to researchers that argue that particularly clinic managers with a physician background prefer that problem identification and decision-making has a scientific approach to facilitate the leadership [27]. However, in previous research, policymakers’ and clinic managers’ ability to include and evaluate the huge amount of evidence on costs and efficiency that needs to be considered in decommissioning processes has been questioned [28]. The strategy in Region Dalarna was to base the problem formulation and decision-making on a scientifically based review, and it seems like this strategy and opportunity appealed to the profession. Researchers emphasise that decommissioning is to be guided by evidence, e.g., obtaining data of existing clinical practice and by using systematic reviews to achieve the best quality of care in the decision-making processes. This requires professionals with competence, engagement, and adequate resources to ensure that evidence drives the process of decommissioning [29].

The clinic managers described the executive leadership team with clinical champions as strong, clear and resolute, and with capability to prioritise the organisation’s overall objectives. The division managers were appreciated for their capacity to lead with integrity and courage, challenge the status quo and having confidence in the professions’ competence to make wise decommissioning decisions. They also had the skills to effectively communicate demonstrable benefits and clarify the relationship between decisions taken and the quality of care. These findings are in line with results in studies that emphasise that the executive leadership team, and in particular the clinical champions, are key influencers during decommissioning processes [12].

Early in the process, the clinic managers and their employees contributed with suggestions on how to decommission services at their clinics. This strategy of early involvement and delegation of large parts of the responsibility for the decommissioning process to the clinic managers and healthcare professions resulted in a feeling of responsibility among the staff.. At the same time, the clinic managers’ and the profession’s formal responsibility forced them to make decisions to minimise risks related to patient safety and quality. This strategy to give the healthcare professions opportunity to be involved and to initiate changes have been reported as successful in previous studies from Sweden [30].

That budgetary pressures are the main driver for decommissioning instead of, e.g., quality and patient safety, have been identified by international experts [4]. However, in our study, the discrepancy between these ideals and budgetary pressures seems to have been adequately balanced by the professions and supported with high-quality data to reduce potential risks to patient safety and quality of care. Researchers also claim that other challenges are associated with decommissioning e.g., that lack of experience on how to carry out decommissioning activities often leads to confusion over roles and responsibilities [2]. According to our results, the executive leadership team, strengthened with legal and analytical expertise, clarified roles and limits of responsibility to facilitate the decision-making processes. This is consistent with results from studies that recommend local healthcare organisations to address the local context, strengthen commitment and clarify expected results and roles in order to succeed with changes as decommissioning activities [6, 13].

Turning to human aspects of change, the clinic managers described the experience of a very demanding, emotionally tiring but personally developing process. The capacity to deal with tensions and very strong emotions during the processes was expressed as an important ability among clinic managers. The clinic manager role as being the one with ability to bridge and soften the negative consequences at staff level during changes have been emphasised in studies [19]. The newly formed divisions created a team feeling that contributed to collegial support among the clinic managers. This collegial support facilitated and to some extent relieved the responsibilities, empowered and strengthened the managers to deal with the outrage that inevitably arose among the staff to some decommissioning decisions. The clinic managers meant that commitment widened, from one’s own, narrow task to include discussions about the entire division’s challenges in the decommissioning process.

In the present study, we identified about three quarters of the 30 subcategories in the decommissioning framework, and we have discussed the most frequently discussed subcategories by the clinic managers. These subcategories were largely the ones that were highly ranked in importance by the international experts in the Delphi study that led to the framework by Robert et al. This indicates that that this framework also has bearing on empirical cases. Although the framework enabled an analysis of important factors based on international experience and can facilitate comparisons of importance in decommissioning processes at different levels in organisations, the categories are to some extent related and difficult to distinguish. A more explanatory description of the categories would have facilitated the analysis to be carried out with greater precision. In future studies, it may be of interest to study if and how the most important factors in implementing decommissioning decisions vary between different levels in healthcare organisations, for example between politicians and clinicians. It may also be of interest to further explore the relationship and interconnections between the three categories and the subcategories. Another good addition would be to investigate the clinic managers’ opinions about all factors through a survey based on the framework by Robert et al.

Furthermore, there are similarities between factors enabling successful decommissioning processes and factors described in frameworks of e.g., de-implementation, implementation and knowledge mobilisation [3, 31, 32]. The latter processes usually starts for the reason that evidence suggests that other interventions could be more beneficial for patients. Although decommissioning may include evidence assessments, it is a broader approach that also addresses resource allocation in the form of re-evaluation and adaption for a multitude of reasons e.g., addressing inequalities, responding to changing demographics, or re-evaluation of resources in order to handle pandemics.

Lastly, this study about decommissioning is timely because healthcare costs are rising in Sweden as well as in other countries, and there is no choice but to address the challenge of how to create more efficient healthcare systems. For example, a mapping of the English NHS showed that as many as 77% of the clinical commissioning groups had decommissioning activities planned [2]. Among the activities reported in the same mapping, the most common type of decommissioning activity was relocation or replacement of a service from an acute to a community setting (28%), removal or replacement of a service as part of reconfiguration of a service (25%) and closure of a service (14%). However, to improve decommissioning policy and practice, it has been identified as crucial to develop a better understanding of how decommissioning programmes unfold in different types of health systems. In this study, the unfolding was studied through the experiences of clinic managers, who are the primary implementers of decommissioning decisions. Our results suggest that when the clinic managers, along with the profession, have the responsibility to identify possible savings and efficiency improvements in the organisation it is likely that quality and patient safety, clinical effectiveness and cost-effectiveness guide the decisions, even if the initial driver was cost/budgetary pressures. Healthcare professionals and in particular, the clinic managers are accountable for patient safety and ethical considerations which becomes inescapable when they have responsibility for both decision and results of the processes. However, to be successful in implementing decommissioning decisions, they need leadership support, convincing evidence and being able to handle strong emotions.

### Limitations

Lastly, a number of restrictions in our study should be mentioned. At first, since health systems differ both between and within different countries, some aspects of the framework may be more or less applicable to particular cases. Subcategories such as, e.g., the reputation of existing providers, availability of alternative services and quality of partnership working with relevant agencies were not mentioned at all in the interviews. This can potentially be explained by the way Swedish healthcare is organised in the regions, but may also be a result of the questions asked during the interviews, which were rather broad in character. However, at the end of each interview, we asked the respondents if there was anything we had missed talking about and if there was something they wanted to add. As all respondents considered that the interview covered what they perceived as most important, we did not revise the interview guide which is an option in interview studies.

Another potential limitation is that the interviews were conducted almost 3 years after the start of the decommissioning process in 2015. There is thus a risk of recall bias even if the decommissioning process was still ongoing at the time of the interviews (i.e., in 2018). However, since some time had passed it is also possible that the clinic managers had had time to reflect on the initial phase of the decommissioning programme and thus be clearer about what was success factors. Third, the clinic managers may also have had an interest in portraying themselves in a favorable way, even though there were no examples in the interviews.

## Conclusions

It has been suggested that failure rates in decommissioning are likely to be higher than in other forms of service change [2], but in this study, we investigated a case of successful decommissioning. The study highlights clinic managers’ experiences by empirically identifying factors that enabled the successful implementation of a decommissioning programme. We conclude that it was crucial to ensure political support and confidence in the clinic manager’s and profession’s ability to handle the decommissioning processes. This was achieved by creating a story supported by evidence that clearly framed the problem and the rationale for change. Furthermore, it was important to have an executive leadership team represented by clinical champions with the ability to prioritise the organisation’s overall objectives. It was also important to involve all clinic managers early in the process, encourage shared responsibility and a fair decision-making process based on evidence and ethical considerations, without compromising quality and patient safety. Lastly, the clinic managers had to handle a process characterised by tensions, resistance and strong emotions, while communication of demonstrable benefits and transparent evaluation of the outcomes of the process was important in the organisation.

## Supplementary Information

**Additional file 1.** Interview guide.

## Data Availability

All data analysed from the interviews are available from the corresponding author on reasonable request.

## Abbreviations

MF: Mio Fredriksson
UW: Ulrika Winblad
LW: Lars Wallin
IBG: Inga-Britt Gustafsson
