# Supplementary material for: Factors that shape the successful implementation of decommissioning programmes: an interview study with clinic managers
Source: BMC Health Serv Res. 2021 Aug 12;21:805. doi: 10.1186/s12913-021-06815-4 (PMC8361631; doi:10.1186/s12913-021-06815-4)
Supplement: Supplementary file 1 — Additional file 1. Interview guide. [file 12913_2021_6815_MOESM1_ESM.pdf]

INTERVIEW GUIDE

Background notes

Profession and years as clinic manager.

What is/has been your role in the work with the implementation of the decommissioning programme?

1a) The start of the process

Who did you perceive as the leading persons in the decision-making process and in the development of the decommissioning programme that began in Dalarna in 2015?

How did you experience the way that the executive leadership team communicated and introduced the need for a decommissioning programme in Region Dalarna?

Who did you perceive as taking responsibility towards employees for the decommissioning decisions announced in 2015? Did you perceive these decisions/suggestions as legitimate?

To what extent did you think the profession was involved in the development of the decommissioning programme, as well as in the further work with priorities and resource allocation?

To what extent were you involved in the decision-making process?

Have your skills and competence, as a clinic manager, been encouraged in the work with the decommissioning programme by the executive leadership team?

Do you perceive that the work with the decommissioning programme has changed over time?

Has it been clear to you what evidence/knowledge base, if any, the decisions were based on?

1b) ***The implementation process***

How have you worked to motivate and gain acceptance for the changes that have been required among your employees?

Please tell me how you worked to involve your employees.

How do you encourage your employees to propose possible changes?

Do you have any example of when the employees' proposals influenced the work at your clinic?

What did their proposals and involvement result in?

Is there anything that has hindered or been particularly difficult in the implementation, initially or now, in the day-to-day work?

What do you think about the pace of change? Has it been speedy, slow or at a reasonable pace?

How would you like to describe your work situation during these last three years?

Did you have access to the support you needed to implement the decommissioning programme in a good way?

Do you have any examples of decisions that you have made that have been crucial to the success of your work?

Do you have any examples of decisions that you regret that made the job more difficult?

What have been the most important factors that enabled the implementation of the decommissioning programme?

3) New experiences and insights

Are the consequences of priorities and resource allocation discussed sufficiently in the region and at your clinic?

On what evidence/knowledge base do you prioritise and allocate resources today compared to how you did prior to 2015?

Has the work with the decommissioning programme affected the view of your own clinic and division or other parts of the local healthcare organisations?

What is your overall assessment of the implementation of the decommissioning programme in the region?

What advice would you like to give a colleague who starts the process of developing and implementing an extensive decommissioning programme in a local healthcare organisation?

4) Closing questions

Is there anything else that you thought about before or during the interview?

Is there anything I haven´t asked about that you wish to add?
